# A review on the production of nanofertilizers and its application in agriculture

**DOI:** 10.1016/j.heliyon.2024.e41243

**Published:** 2024-12-14

**Authors:** Birara Melku Ayenew, Neela Satheesh, Zemenu Birhan Zegeye, Desalegn Adisu Kassie

**Affiliations:** a Faculty of Chemical and Food Engineering, Bahir Dar Institute of technology, Bahir Dar University, P.O. Box, 26, Bahir Dar, Ethiopia; b Department of Food Science and Technology, School of Health Sciences, Amity University, Mohali, Punjab, India; c Colleges of Agriculture and Environmental Sciences, Department of Animal Sciences, Institute of Biotechnology, Bahir Dar University, and P.O. Box 5501, Bahir Dar, Ethiopia

**Keywords:** Nano-fertilizer, Nanotechnology, Nanoparticle, Crop production, Soil fertility

## Abstract

Due to the rapid rise in the worldwide population, the need for food is expanding constantly. To boost agricultural productivity large amounts of synthetic fertilizers are used. However, the extensive use of these synthetic fertilizers leads to various environmental and health problems. Nanotechnology (NT) offers significant improvement in the fertilizer fabrication with optimal chemical compositions, enhances nutrient usage competence, reduces environmental influence, and increased plant productivity. Nano fertilizers (NF) are nanomaterials (NM) that contrast significantly from their corresponding bulk materials. The concept of NF technology is highly innovative, utilizing physical, chemical, and biological methods for formulation. Additionally, the precise application and targeted delivery of nano-fertilizers ensures a more sustainable and accurate approach to agriculture. The purpose of this review is to provide an overview of the various types of nano fertilizers and their synthesis by different methods. In addition, applications of the NF and advantages are also given over the conventional pesticides. Information is also given on the current applications and challenges of the NF. NF are more effective and efficient than conservative fertilizers. The use of NF is expanding owing to the positive influence on nutritional superiority and stress resistance in plants.

## 1 Introduction

Worldwide agricultural practices are intensively utilizing a huge number of agricultural inputs to realize higher food production. Fertilizers are important agricultural inputs in enhancing agriculture crops yields and quality mainly. Current agricultural systems cannot meet the growing demand for food without the extensive use of fertilizers [1,2]. However, conventional fertilizers are inherently inefficient for plants to effectively absorb and utilize nutrients. Over the last 60 years, the usage of conventional fertilizers has portrayed a fundamental function universally in cumulative produce yields and continuing sufficient food resources. Numerous findings relating to the prolonged utilization of chemical fertilizers and their influence on agroecological techniques are readily documented [3]. However, exceeding the higher quantities of the fertilizers restrains several issues, especially environmental pollution [4,5].

Currently, agriculture faces extensive gamut of summons across the globe like nutrient deficit in soil, stagnation in crop harvests, waning soil organic substance, decreased water obtainability, decreased cultivable land owing to suburbanization and land dilapidation, and employment deficiencies [3]. The relevance of NT is spreading exceptionally, and research findings are incessantly recommended naive methods and appropriate resources for crop production and organization [6]. The NF are eco-friendly fertilizers with the hypothetical to surge the relevance proportions of fertilizers and ease the deficit of nutrients commencing it, predominantly P and N_2_ [7]. It offers regular and monitored delivery of nutrients to specific locations, they assist in stopping the contamination of water resources and the atmosphere [8].

NF are one of the most important crop production inputs which are integrated or reformed from conventional fertilizers or from bulk materials being industrialized using material with the nano scales (1–100 nm). The wide-range surface vicinity of nanoparticle efficiently retains profusion nutrients and staggered release to enable realization of nutrients corresponding with the crop essentials. NF has a small size and high surface area that helps to reach wider sites or target areas of plants’ body to enhance different metabolic activity and increase its responsiveness with additional complexes and solubility in other solvents like water. Owing to the diminished size, both physical and chemical possessions of nano materials are different from that of bulk or conventional ones in similar substances. NF particles are smaller in size as compared to pores of plant roots or leaves that help to penetrate and enter to plants easily improving nutrient use efficiency and concurrently it can result in noteworthy decrease in the quantity of substances that need to be functional [9].

The focal role of NT in agriculture is to formulate and design fertilizers for regulating and releasing of nutrients based on the needs of production. Very small size of the prepared mineral micronutrient can advance solubility and dissemination of inexplicable nutrient in soil, diminish soil captivation, complex and upsurge bioavailability and nourishment competence and acceptance ratio of the soil nutrients in crop intervention. NF are extremely efficient for accurate nutrient administration in exactitude food production by saving fertilizer resources and increasing yields of plant crops through optimal nutrient management. NT also helps agriculture by reducing environmental pollution not only by reducing fertilizer use but, reduced the use of substitute (renewable) energy provisions like feather waste and filters or catalysts and clean-up prevailing contaminants after production of pesticides and conventional fertilizers using the nanoparticles (NP) and nano capsule [9,10].

Nano particles which are biologically originated polymers of carbohydrates and proteins with subdued impact on human wellbeing and ecology. Hence, NT has disparagingly imperative for encouraging the advance of ecofriendly and justifiable agriculture production by providing feasible nano designed ingredients as fertilizer haulers or staggered proclamation vectors for edifice of “smart fertilizer” as new facilities to augment nutrient use competence and decrease costs of environmental safety [11].

By considering the advantages over the NF, this review aims to provide data on the different aspects like preparation of NF, types of NF, NF role in crop nutrition, the status and challenges are summarized.

## 2 Role of nano technology in preparation of plant fertilizer

Food is one of the basic needs that humans can deserve, but food security is underneath profound threat around the globe. Agriculture aspects face many confronts, such as diminished crop yields due to both biotic and abiotic pressures, involving nutrient shortage and ecological pollution [12]. Rapidly increasing population made to prepare higher food requirements and the constant drop in farmland per capita persuades continuous push for using fertilizer application for crop production to sustain food demand. Fertilizers are agricultural inputs which contain chemical elements and play a crucial role globally by improving growth and productivity of plants by enhancing the natural fertility of the soil [13].

The prompt and rapid progression of scientific revolutions and use of chemical fertilizers fronts to insightful underlying fluctuations in the field of agriculture. However, spending substantial quantities of fertilizers cannot secure appropriate solutions. Likewise, such fertilizers are not safe for the environment, instead the usage of significant amounts of chemical products directs problems such as contamination of soil and water supplies, modification of soil organization and destruction [14].

Currently, NT is progressively applied to different areas by formulating different materials at nanoscale, especially NF to replace chemical fertilizers offers promising applications for precision agriculture farming system [11]. Consequently, the agricultural segment is presently observing forward to contemporary technologies that assistance to upsurge crop efficiency, resistance nutrient up takes efficiency of the crop, and able to minimize environmental impact by controlling excessive nutrient release. So that new insight and imperative need to create smart raw material that can methodically modify for a control release of nutrients to specific targeted sites of plants in agriculture practice [15]. According to Hossain et al. (2020) nanoscale science and nanotechnology provide innovative and improved resolutions to various terrific argues opposite in agriculture and the civilization currently and in the future [16]. This technology is the sixth truly revolutionary technology introduced in the modern world to an original methodology by using equipment and apparatus accomplished of employing physical as well as chemical possessions of an ingredient.

## 3 Types of nano fertilizers

NF contains nanosized elements, which plants can absorb and enhance crop produce. These NF produced by the application of NT with probable claims in agriculture, but their taxonomy showed contradictions. Conversely, some definitions also comprise additional produces, such as nanoscale approach systems and nano-biosensors. To illustrate, NF are categorized as a subsection of NT and as a type of fertilizer. This ambiguity has commanded to a lack of simplicity on the definition and classification of NF, which may lead to confusion when assessing their use and potential compensation.

**Fig. 1:**
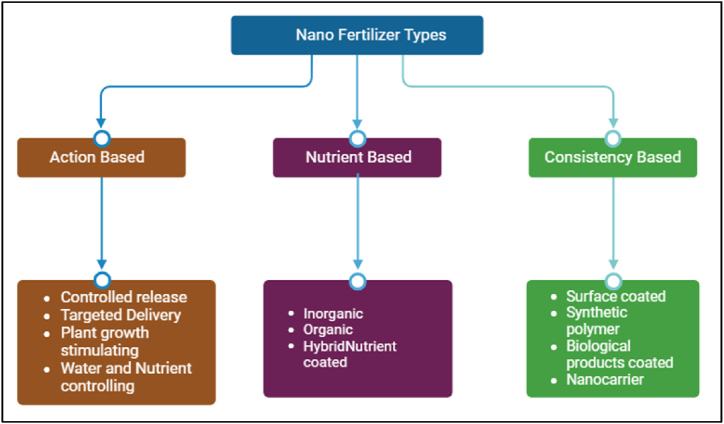
Classification criteria for nanofertilizers.

NF furthermore be categorized on the raw material considered in the preparation. To illustrate, certain NF are prepared with carbon nanotubes, in contrast some are fabricated with polymers or metals. Each NF has dissimilar assets and can have broader impacts on plants. NF are mostly categorized based on the nutrients they carry, the actions they perform, and their consistency (Fig. 1). Knowledge on the nature of NF is fundamental to obtaining the best application approach. NF can be utilized to plants through foliar, water, and soil application [17].

NF in terms of action-based segregated in to controlled-release, targeted transportation, plant growth-stimulating and water and nutrient loss organizing. Whereas nutrient based NF are further classified into hybrid, nutrient coated, inorganic, and organic. The consistency-based NF is divided into surface coated, synthetic polymer, organic products coated and nano carrier. These groundbreaking NF bargain a range of assistances, such as enhanced nutrient utilization, controlled nutrient release, embattled nutrient delivery, improved plant growth, and lower nutrient loss, making them valuable for sustainable agriculture production [18]. Based on the nutrients, the nano-biofertilizer is divided into the following types.

### 3.1 Nitrogen nanofertilizer

Nitrogen (N) is a critical component needed for the survival of plants, which is limited to plants at various conditions, and it is highly reactive with other chemical agents (e.g., NH_3_, HNO_3_, cyanides, and organic nitrates). Nitrogen is a nutrient that needs to be applied in agriculture land to improve crop production. It is available in the market in both solid or liquid state as NH_3_, CO(NH_2_)_2_ and NH_4_NO_3_ [19]. Plants unable to utilize N from the air/atmosphere, this nutrient should apply in soil in the form of NH_3_, HNO_3_ and CO(NH_2_)_2_, by adding these compounds in the soil reacts with H_2_O and release fertilizer as NH_3_ ions. This is further nitrified by the action of the bacteria to nitrate ions which are required for crops [19].

Farmers use overdoses of the fertilizers in certain situations by expecting higher amounts of the harvest. However, these practices impose the risk to the soil, it alters the organization and composition of the soil [20]. Leached fertilizers advance to eutrophication into water bodies. Some researchers recommended nitrification inhibitors, stabilizers, additives with the N fertilizers to reduce the loss of NH_3_ developed throughout consequence of the fertilizers with the soil and H_2_O [20]. This approach facilitates the better absorption of the fertilizers into the soil and facilitates enough time to react with the appropriate H_2_O [21]. Researchers also reported for proper soil absorption, N fertilizer should be applied in the form of ammonium ions. In contrast, nitrates are complicated to assimilate the soil or organic manure particles. In the case of ammonia, they escape easily into the surrounding atmosphere and, they need special farm practices [22]. Hence, researchers are suggested to practice the tilling before application and similarly applying this part in the internal layers with adequate water. Urea is the mostly used N fertilizer as it contains higher concentrations of the N, it is highly compatible with other micro or macro nutrients, convenient for handling and application [22].

The N is not accessible as they are negatively charged. Nitrate generally showed very poor affection to the soil particles surfaces, thus cannot willingly absorb on to the soil. To address the issues related to the N fertilizer, researchers have reported urea coating with the polyolefin resin, neem, sulfur for staggered release of N release [23]. Nevertheless, the limited release of fertilizers is frequently costly, and takes more time to reach plants. Nitrogen reduction can also be diminished by the application of the positive ion exchangers to stagger NH_4_^+^ liberation. The memory and timely distribution of needed nutrients by zeolite increases whole crop produce [20].

Zeolite is a mineral asset, secure to the soil and tasks well to safeguard accessible soil nutrients, for competent crop fabrication and decline in ecological hazards correlated with agricultural performance [24]. Natural zeolite, encompasses greater than 50 minerals fundamentally composed of alkali and alkaline earth alum inosilicates, has been obtained into NF in current times. This is principally owing to its convenience and economic benefits, and it has been utilized in agriculture [24,25]. In addition, zeolite is a smart transporter and controller of conventional fertilizers. Zeolite carries N and K, indicating decreased concentration measures with improved yield [26]. Zeolite addition with humus enhances the productivity of crops. In addition, zeolite nanocomposites of N, P and K are in additional nutrients and micronutrients like amino acids, mannose, Ca, Fe, and Zn. One disadvantage of zeolite is its inability to adsorb anions, thus it is enhanced in this perspective with biopolymers [24]. The zeolite can fix ammonium and allow staggered release of the N [23]. Various studies reported that zeolite is a source of the N which upgrades N usage efficiently. The surfactant-modified zeolite (SMZ) by hexadecyltrimethyl ammonium as fertilizer carrier showed wonderful results to sorbent nitrates. Similarly, surface modification with hexadecyltrimethyl ammonium and dioctadecyldimethyl ammonium awfully increases the sorption and slow release of nitrate [23]. NF distributed nutrients up to 1200 h while, traditional fertilizer can be available for 300–350 h. Finally, it is concluded that, zeolite as NF ideal strategy to boost nitrogen use effectiveness [27].

### 3.2 Phosphorus nanofertilizers

Phosphorus is a prime compound in plant structure and has catalytic role in numerous vital biochemical responses in plants. This mineral has a distinct role in cell division, formation of new tissue and energy transfer and storage in the plants. P based complexes are part of the energy pool, they are formed in the part of the photosynthesis and carbohydrate-based energy synthesis. Later, they are used for plant growth and breeding [28]. The appropriate concentration of the P in the soil advocates faster root growth development and faster maturity of plants. Conversely, poor P can enhance the sugars formation in plants and develop reddish purple pigments like anthocyanins [28]. Different authors observed the solubility and exchange of cation in the mixture of rock P, NH_4_^+^ and K^+^ saturated clinoptilolite for staggered release of P by dissolution and ion-exchange mechanism [23].

Saturated clinoptilolite with NH_4_^+^ and K^+^ (monovalent cations) intensified the solubility of rock P. The competence of P varies from 18 to 20 % in a year, remaining 78–80 % converts to the soil P pool, that will be used by the plants later [22]. Researchers reported the liberation of P from fertilizer-loaded unmodified zeolite, SMZ and solid KH_2_PO_4_. Results reported SMZ showed best fertilizer carrier properties for staggered release of the P (H_2_PO_4_). The reaction of the P is presented in the following equation.

(P-rock) + (NH_4_– zeolite) = (Ca zeolite) + (NH_4_) + (H_2_PO_4_)

Zeolite takes Ca^2+^ from the rock P and can release equally P and NH_4_. Contrasting the leaching of highly soluble P established equilibrium [22]. P is also released from rock P by reducing soil pH as NH_4_ is converted to NO^−^_3_.

Zeoponic is a nutrient transfer scheme from PO_4_^3−^ and other nutrients released by controlled dissolution. Essentially, the procedure is a dissolution and ion exchange reactions. The assimilation of nutrients from the soil to plant leads to the formation of suspension and ion exchange reactions, pulling away nutrients which are required. The zeoponic approaches enhance nutrient preservation, diminish environmental nutrient depletion, and reduce fertilizer needs by proving reasonable nutrient allocation in the plant root zone [22]. NF suggests that surface modified zeolite could be potential strategy to promote phosphorous use efficiently which hardly exceeds 18–20 % in conventional system [27].

### 3.3 Potash nanofertilizers

K is vital to plant metabolic activities like photosynthesis, protein synthesis, ionic balance, regulation of plant stomata and water use, activation of plant enzymes and many other processes. Duan et al. (2023) reported staggered release of K trapped in zeolite [29]. Similarly, other scientists developed the cation exchange competence of the nano clays when replacing the Si_4_^+^ with Al_3_^+^, this enhanced negative ionic nature of the lay lattice due to the ammonium, sodium, calcium, and potassium exchanged with other ions. K fertilizer is absolutely implicated in photosynthesis, it contributes to regulation of the stomata. The polyacrylamide-based pellets are reported for the slower release of K fertilizer and available for 35–40 % of crop productivity. The effectiveness of N, P and K have maintained stable at 30–35 %, 18–20 %, 35–40 %, respectively. To avoid multi-nutrient failings, unfair fertilization, minimal fertilizer utilization and reduced soil organic manure is supporting to develop a nano-based fertilizer for smart provision of nutrient to directed site.

The use of NF (nano K) in foliar spray (640 mg/ha (40 ppm concentration)) results in higher yields of cluster bean and pearl millet in arid climates. The research data proposes that stable fertilization can also be delivered through a nanotechnological approach to meet crop demand and fertilizers encapsulated in nanoparticles will enhance the uptake of nutrients [30].

Currently, commercial NF are available for agriculture applications. For instance, eco-friendly nano-leucite form of K, Al_2_SiO_6_ showed staggered liberation of nutrients, outstanding cation exchange capability, nutrient extending capability can be enhanced by salt obstruction. Similarly, increased plant growth and enormous stem diameters were reported by the nano based K fertilizer application at concentration of 300 kg. ha^−1^. Researchers evaluated the efficacy of P–K NF carried by zeolite in plants and conducted in *vitro* studies for 30 days 47 on *Ipomoea aquatica*. The findings showed K and P NF are given better results in comparison with the conventional fertilizers [3].

### 3.4 Calcium nanofertilizers

Ca is one more critical plant nutrient, has a role in cell wall synthesis and plant growth regulation. Ca is a structural part of cell wall and provides structural rigidity by facilitating the crosslinks in the pectin-polysaccharide matrix. Ca facilitates the protection and resistance to diseases (bacterial and viral) in plants. Application of nano-Ca successfully enhanced the development and physical growth of the plant. Very limited studies are reported on the effects of nano-Ca on plants showed enormous potential as fertilizers [3].

### 3.5 Magnesium nanofertilizers

Mg is a micronutrient shows a cardinal role in plant enzymes like ATPase, RNA polymerases, etc. The deficiency in Mg significantly influences the rate of photosynthesis. Researchers reported that the foliar treatment of Mg enhanced the seed yield and protein substance in plants. In addition, application of Nano-Mg in tomatoes at the rate of 0.1–1.0 % (@ rate 0.5 mg. L^−1^) enhanced the photosynthesis rate, growth, and yield and similar results also observed in the cowpea also. These Nano-Mg also showed the rises in seed weight, plasma membrane stability, and chlorophyll in different. Other reports stated the 13.4 % greater yield of black-eyed pea, by the application of 0.5 g L^−1^ of Fe salt and 0.5 g L^−1^ of Mg-NP in parallel with the controls [3].

### 3.6 Zinc nanofertilizers

Zn is a micronutrient that has special responsibility in tryptophan formation and photosynthesis, cofactor in dehydrogenases, aldolases, isomerases. Researchers showed the influence of the Zn-NP on *Punica granatum* L. *cv. Ardestani* plants. Similarly, zinc–boron NP (Zn-B-NP) at 60 and 120 mg/L results showed the fruit yield was suggestively higher (63–66 fruit per tree) as compared to the control (51 fruit per tree). The nutrients Zn possess a special role in the pollen formation, tube growth, and enhanced flowering and fruits with the bigger sizes [31].

### 3.7 Nano porous zeolites

Nano porous zeolites (NPZ) have broad structural attributes such as elevated surface area and substantial aperture capacity which can improve problems with the natural zeolites as it contains microporous structure. Various innovative synthetic methodologies were employed for the NPZ such as post-demetallation, soft and hard-templating, and dual-pore-formation were used. The NPZ reported application in isomerization, cracking, alkylation and oxidation [27]. Zeolite is a critical class of crystalline alumino silicate minerals with microporous compositions. The microspores are very identical in size and shape, which can differentiate particles based on the size and shape [3,27]. Zeolites possess a special role in the molecular selective adsorption (based on size/shape), separation methods involved in the host–guest chemistry. The structural (crystalline) arrangement of zeolites imparts very high thermal, hydrothermal, and mechanical stability, these properties permit zeolite to be used in several catalytic conditions beneath the harsh reaction circumstances [3].

## 4 Preparation methods of nanofertilizers

**Fig. 2:**
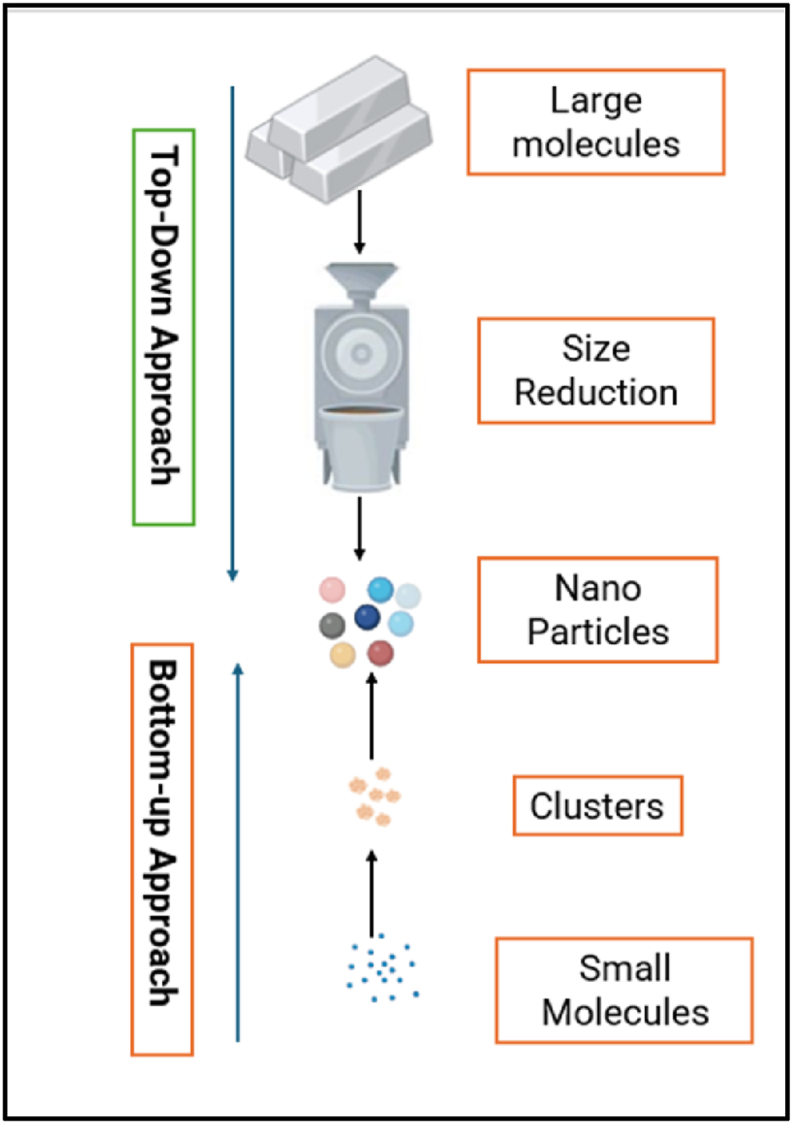
Top down and Bottom-up approach of the nanoparticle synthesis.

According to the Royal Society and Royal Academy of Engineering, NM can be prepared through either top-down or bottom-up (BU) approach depending on size reduction from bulk materials and materials which are synthesis from atomic level, respectively (Fig. 2). Especially, NF are synthesized by invigorating nutrients individually or in mixtures onto the adsorbents with nano-dimension through top-down or BU. These nutrients are encumbered to cationic nutrients (NH_4_^+^, K^+^, Ca^2+,^ Mg^2+^) and afterward superficial alteration for anionic nutrients (NO_3_^−^, PO_4_^2−^, SO_4_^2−^) [32].

### 4.1 Top-down approach

A top-down approach method includes various types of milling or attrition, chemical methods, and volatilization of a solid trailed by strengthening of the volatize component. Additionally, the tiny manipulations of a small number of atoms or molecules fashion elegant designs, using mechanical or physical approaches such as grinding, milling and crushing to generate NP. This procedure is used to yield nanocomposites and nano grained bulk materials such as metallic and ceramic NM in distribution in which size reduction from cm to nm level [32].

### 4.2 Bottom-up approach

The other methods for preparing NM are BU by constructing various molecules starting from a homogeneous solution or gas and building up the nanoparticle by self-assembling in parallel stages [33]. This method can create more complex structures with uniform control size, shapes and size ranges of NM by using atoms or molecules [32].

It is very important to synthesize NP in specific scale, morphology. Therefore, the minimal procedure for organizing nanostructure is advocated by caustic compounds, such as reducers, surfactant materials, polymers enveloping agents, many organic solvents etc., [34].

Thus, numerous compulsory constraints are insisted to develop NP manufacturing procedures that are ecofriendly without using harsh complex resources in manufacturing procedures. Therefore, many researchers reported the role of several microorganisms in preparation of inorganic nanomaterial structures (either internal or external) [35].

The microbial mixture of NM like silicon oxide, metal and metal blend is acknowledged as a biological mineralization process. Employing ambient conditions in bio-mineralization processes or NP biological synthesized products are reported [36].

### 4.3 Chemical methods

The most typically employed technique for NP preparation is chemical reduction. BU approach is a colloidal technique which is attracting for the assembly of NP. In the BU approach allowing the formation of NP with metal by formation of chemical attachments [33].

The chemical methods commonly used are condensation, hydrolysis, and thermal decomposition of nutrients in solutions and this process leads to sol formation. However, the gel is formed by hydrolysis or condensation due to the higher viscosity. The element proportions can be influenced by the precursor intensity, temperature, and pH ranges maintained [37]. The sono-chemical merging with Cu salt in the addition of Pd and H_2_O was also reported for the NP developments [38,39]. Furthermore, in sedentary gas solidity performance NP are constructed via vanishing of metals obtained in zero element gas, had been broadly treated to yield fine NP. The Cu-NP is produced by exposing the metal to zero element gases (Argon, Helium and Neon) resulting in metal vaporization. The vaporized metals immediately lose energy by cooling (by liquid N) of the vaporized atom to form NP of 2–100 nm size [40]. NP synthesis by hydrothermal methods requires a wide range of temperature (room to very high temperatures). However, NP created in hydrothermal process may be unstable at greater temperatures [41].

### 4.4 Physical approaches

The physical approach in the fabrication of NP involves thermal breakdown, dissemination, electrolysis compression and application of laser. In case of the size reduction, various types of mills are considered to reduce the size of the large size particles to the nanosized particles. For instance, mills like planetary, vibratory, rod, tumbler, for the formation of NP of Co, Cr, W, Ag and Fe. However, the mill operation factors like operation speed and duration of milling are significantly influencing the size of the NP [42,43]. In case of higher temperature applications, molten state of metals is allowed to detain in a glass by maintaining the higher velocity with turbulence. In the process, amorphous solids-metallic glasses are formed by cooling rapidly [44,45]. Similarly, a melted stream of Cu-B and a heated stream of Ti forms NP of TiB_2_ [46,47]. The usage of the laser is another constantly used method for the preparation of the NP. The properties of the NP are based on various factors like laser, pulses, type of solvent, pulsing time [48,49]. Table 1 composed the synthesis of different NP using ball mill methods (Table 1).

**Table 1:** Synthesis of diﬀerent nanoparticles (used as fertilizer) using different physical methods.

Types of NP	Size of metallic powder	Ball mill type	Mill speed	Milling duration	Characterization techniques	Size of NPs	References
ZnO	0.6 nm	Horizontal Oscillatory mill (retsch, PM400)	–	2–50hr	XRD, TEM.SEM, HEBM	TEM 30-20 nm SEM 30 nm	[32]
ZnO	500 nm	Horizontal Oscillatory mill (Retsch, MM2)	–	1hr	XRD, SEM, PL	110-50 nm	[3]
ZnO	ZnO powder	–	–	40hr	TEM, UV–Vis, XRD	5–10 nm	
CuO	Cupric acetate	Ultrasonic wave assisted ball milling	256r/min		XRD, TEM, UV–Vis and fluorescent measurement	20 nm	
CuO	60um	–	450 rpm	0–60hr	XRD and TEM	11 nm	
TiO_2_	>0.5 mm	Szegvari Attritor	400 rpm	10hr	XRD, TEM, UV–Vis spectroscopy	10–20 nm	[32]
TiO_2_	TiOSO_4_ xH_2_SO_4_ and NaCl powder	–	400 rpm	4hr	XRD and TEM	Anatase 28 nm Rutile 37 nm	
TiO_2_	Fine Ti and Fe powder 40um	Planetary ball millPM400/2 (Retsch, Germany	0rpm 250 rpm 300 rpm 350 rpm	5hr 6hr 5hr	XRD, SEM, EDX and UV–Vis	22 nm 16 nm 14.91 nm 32.69 nm 14.84 nm 30.47 nm 14.76 nm 29.67 nm	[3]
Ag	Ag_2_O Powder (5-40μm)	–	–	95hr 22hr		14 nm 28 nm	

### 4.5 Biological methods

**Fig. 3:**
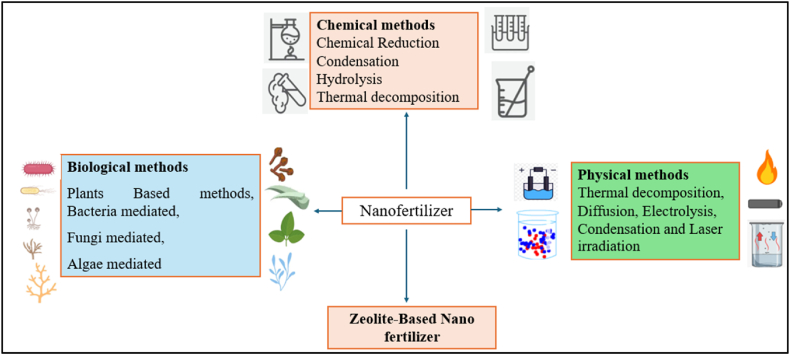
Nanofertilizer synthesis by different methods.

Researchers reported the NP synthesis by the application of microbial process have expanded additional consideration due to cost-efficiency and environmentally sustainable process. Microbes synthesize NP by extracellular or intracellular biosynthesis (Fig. 3). Microorganisms have the capability to synthesize certain enzymes like reductases, which are highly used in the bioremediation or storage of certain metals, these capabilities are taped by the scientists for the synthesis of NP. Researchers have developed Cd-S NP by the application of the *Klebsiella pneumonia* [50,51]

#### 4.5.1 Bacteria mediated NP synthesis

Researchers reported the NP synthesis by various bacteria and actinomycetes which embrace the intracellular fabrication method (Table 2). The microbial pellet attained from centrifugation is patronized with the addition of the metal salts with various strengths and allowed for incubation in ambient environments (temperature and pressure) in dark. However, extracellular synthesis of NP is carried out by addition of metals in the bacterial liquid culture. After incubation centrifugation was carried at 8,000X to separation of the NP [33].

**Table 2:** Synthesis of diﬀerent nanoparticles using diﬀerent bacteria used in agriculture.

S. No	Types of NPs	Name of Bacteria	Size	Morphology	References
1	Silver	*Lactobacillus casei*	20–50 nm	Spherical	[32]
2	Silver	*Bacillus cereus*	20–40 nm	Spherical	
3	Gold	*E. coli*	8–50 nm	Spherical	
4	Gold	*Bacillus subtilis*	5–50 nm	Hexagonal-Octahedral	
5	Iron oxide	*Magnetospirillum magneto tacticum*	47 nm	–	
6	Iron oxide	*Aquaspirillum magneto tacticum*	40–50 nm	Octahedral prism	

#### 4.5.2 Fungi mediated NP synthesis

NP can effectively be manufactured by eukaryotes like fungi (Table 3) due to the various advantages. The attributes like growth in the under controlled circumstances, highly tolerance to the mutations and wonderful external synthesis of the enzymes and proteins which are useful in the NP formations [34,33].

**Table 3:** Synthesis of diﬀerent NP using diﬀerent fungi.

Type of NPs	Fungal strain	NP Size	Shape	References
ZnO	*Aspergillus strain*	50–120 nm	Spherical	[[32], [34], [33]]
ZnO	*Candida Albicans*	15–25 nm	Hexagonal	
ZnO	*Aspergillus terreus*	29 nm	Hexagonal	
Ag	*Trichoderma viride*	2–4 nm	Spherical	
Ag	*Fusarium Axysporum*	5–13 nm	Spherical	
Ag	*Arthroderma Fulvum*	20.56 nm	Spherical	
Au	*Collectotrichum* sp	8–40 nm	Spherical	
TiO_2_	*Aspergillus flavus*	62–74 nm	Spherical	
Pt	*Fusarium axyporum*	70–180 nm	Rectangular, triangular and spherical	

The NP synthesis takes place intracellularly in the fungal vegetative bodies when grown in the metal salt broths incubated by vigorous shaking for 24 h. Similarly, researchers also reported the application of the dried fungal mycelium in the formulation of NP. In the dried process, the freeze-dried mycelium is treated with metal salt dilutions and allowed for incubation for 24 h for the NP formulation [33].

#### 4.5.3 Algae based NP synthesis

Researchers reported different methods in NP synthesis by algae. For NP preparation extracted and washed algal bodies are treated with metal salts at ambient pH and temperature conditions and incubated in the absence of light. Further, cell-free culture contained NP incubated at −20 °C. In the freezing process NP attain higher density structure and settled at the bottom [33].

### 4.6 Synthesis of NP using plant extracts

Plant based components showed a very important role in the biosynthesis of various NP and these methods are considered as green synthesis. Various researchers have reported the usage of the geranium herb (*Pelargonium graveolens*) leaves in the synthesis of Au NP [33,52,53]. Usually, plant-based biomolecules possess a specific responsibility as bio reductants, which have the capacity to transform the metal ions into NP (Table 2). Many plant extracts like *Phyllanthus amla*, geranium, coriander, *Aloe vera* and neem tea tree leaves, Alfalfa and *Capsicum annum* were reported for the greener production of NP. The reducing factors (e.g., alkaloids, phenolic compounds, terpenoids, flavonoids, and coenzymes) are concerned in reduction of the metal ions involved as colloids. Different researchers (Table 4) targeted preparation of Ag and Au NP due to their therapeutic and ecological application [34,33].

**Table 4:** Synthesis of diﬀerent nanoparticles using diﬀerent plants as nanofertilizer.

S. No	Types of NPs	Plant type	Part of plant used	Particle size	Usage	References
1	Gold and Silver	*Aloe vera*	Leaf	10–30 nm	Optical coatings and cancer hyperthermia	[[32], [54]]
2	Gold	*Syzygium Aromaticum* (clove buds)	–	5–10 nm	Detection and destruction of cancer cells	
3	Silver	Lemon	Lemon extract	<50 nm	–	
4	TiO_2_	*Aloe vera*	Leaf	60 nm	–	
5	TiO_2_	*Psidium guajava*	Leaf	32.58 nm	–	
6	TiO_2_	*Annona squamosa*	peel	23 nm	–	
7	ZnO	*Albizia lebbeck*	Stem bam	66.25 nm	Antimicrobial, antioxidant	
8	ZnO	*Trifolim pratense*	Flower	100–190 nm	–	
9	ZnO	*Azadirachta indica*	Leaves	40 nm	–	
10	Cu	*Tinospora cordifolia*	Leaves	50–130 nm	Catalytic degradation	
11	Cu	*Citrus medica* Linn	Juice	33 nm	Antimicrobial	
12	Cu	*Ixoro coccinea*	Leaves	80–110 nm	–	
13	Fe	Pomegranate	–	100–200 nm	–	
14	Fe	Plantain	peel	<50 nm	–	

Various physical and chemical techniques are utilized for synthesis of the NP. Furthermore, fabrication is managed at aggregate temperatures which requires a large quantity of overheating. While using the thermal decomposition process, terribly extreme temperatures are used [33]. NP by biological methods (application of bacteria, fungi, actinomycetes, yeast, algae, and plant extracts) provide a good variety of different resources for manufacturing NP [55]. Rapid metal ions reduction in ambient conditions is the prime advantage for considering the biological methods for NP synthesis. For instance, Au-NP prepared by *Aspergillus niger*, find in 2 h [33].

### 4.7 Zeolite-based nano fertilizer

Zeolites are microporous, aluminosilicate-based materials used in agriculture for absorbing and retain water, nutrients, and other organic compounds [56]. The formation of the zeolite-based NP combined with several supplementary combinations to generate fertilizer. These NF are small, allowing them to penetrate deeper into the soil and deliver nutrients to a plant root zone more efficiently [57,58]

As already mentioned in the previous sections, zeolites can hold minerals and act as the reservoir for storing and releasing nutrients over time constantly for longer durations. In addition, zeolite-NF can modify the specific nutrient needs of a crop, ensuring that the required nutrients only can provide to the plant, this approach can be used to reduce the expenditure of fertilization. The type of NF application varied based on the soil type, crop requirements, and environmental concerns. Similarly, Chitosan- NF attracts various agricultural applications due to the benefits of staggered delivery, biodegradability, and ability to adapt. However, zeolite and chitosan- NF can be mixed easily with supplementary nutrients and agrochemicals and facilitating the adaptable solution for sustainable farming practices. Properties of the natural zeolite and nano porous zeolite are presented in Table 5.

**Table 5:** Average particle size distributed zeta potential natural zeolite, nonporous zeolite and fertilizer formulations at 1:1 ratio.

Attributes	Natural Zeolite	Nano porous zeolite	References
PH (1:6.25 ml ratio)	9.6	8.14	[59]
ECe(dSm^-1^) (saturated paste)	0.17	0.06	
Moisture (%)	10	12	
Bulk density (Mgm^−3^)	0.57	0.50	
Particle density (mgm^−3^)	0.66	0.39	
Pore space (%)	34	45	
Total organic carbon (%)	1.9	1.03	
Total Nitrogen (%)	0.02	0.03	
Total phosphorus (%)	0.06	0.02	
Total potassium (%)	0.09	0.02	
Calcium (%)	5.25	–	
Magnesium (%)	6.03	–	
Silica (%)	4.78	1.49	
Aluminum (%)	1.02	1.59	
CEC (cmol)(P) Kg^−1^)	100	106	
Inorganic carbon (%)	28	45	

### 4.8 Encapsulation of nanofertilizer

Nanocapsules (NC) are microscopic materials compiled of organic or inorganic substances used to encapsulate and transport fertilizers to the target [60]. NC prepared from various biological based polymers, lipids, silica, metal oxides, and carbon nanotubes [61]. NC creates defensive layers and facilitates the staggered release of the nutrients for longer durations. This encapsulation allows higher competent fertilizer use, excellent control over its application, retaining nutrient stability, and providing a controlled release of the fertilizer to the crops. The encapsulated NP ensures the nutrients are released appropriately at the required time. In addition, limited release of the nutrients from the entrapped nutrients reduces the possibility of leaching. Various authors showed the prospective advantages of NC-based NF in cultivating nutrient function and successful crop yields. For example, Salam et al., reported by application of the NC ZnO-NP given higher maize yields. Similarly, it is also reported that the increased rate of photosynthesis and plant growth [62]. Kottegoda et al. [61], encapsulated urea NF facilitates excellent delivery of nitrogen and improves plant growth as compared to the commercial urea. The nanocomposite mixture was then encapsulated into the hydroxyapatite using ultrasonicator to enhance the staggered release of N to the plants [63,64].

## 5 Nano fertilizer role in crop nutrition

For sustainable agriculture, the application of innovative types of nutrients or fertilizers is one of the encouraging methods. These novel fertilizers are useful in increasing plant growth to satisfy the food demand and environmental concerns (Table 6). NF can provide single or combined nutrients in agriculture to improve plant growth as compared with the traditional fertilizers [32] (Table 6).

**Table 6:** Application of Nanofertilizer and their effect on productivity of different crops under varying climatic conditions.

Nano fertilizers	Crop	Yield increment (%)	References
Nano fertilizer + Urea	Rice	10.2	[21,65,66]
Nano fertilizer + Urea	Rice	8.5	Kim et al., 2020.
Nano fertilizer + Urea	wheat	6.5	[21,66]
Nano fertilizer + Urea	wheat	7.3	64
Nano-encapsulated phosphorous	Maize	10.9	[21,66]
Nano-encapsulated phosphorous	Soybean	16.7	[66]
Nano-encapsulated phosphorous	wheat	28.8	[32]
Nano-encapsulated phosphorous	Vegetables	12.0–19.7	[21,66]
Nano chitosan-NPK fertilizers	wheat	14.6	[65]
Nano chitosan	Tomato	20	[32]
Nano chitosan	Cucumber	9.3	[32]
Nano chitosan	Capsicum	11.5	[65]
Nano chitosan	Beet-root	8.4	[21,66]
Nano chitosan	Pea	20	[32]
Nano powder of cotton seed and ammonium fertilizer	Sweet potato	16	[32]
Aqueous solution on Nano iron	Cereals	8–17	[65]
Nanoparticles of ZnO	Cucumber	6.3	[32]
Nanoparticles of ZnO	Peanut	4.8	[65]
Nanoparticles of ZnO	Cabbage	9.1	[21,66]
Nanoparticles of ZnO	Cauliflower	8.3	[65]
Nanoparticles of ZnO	Chickpea	14.9	[32]
Rare earth oxides nanoparticles	Vegetables	7–45	[32]
Nano siliver + allicin	Cereals	4–8.5	[21,66]
Iron oxide nanoparticles + Calcium carbonate nanoparticles + peat	Cereals	14.8–23.1	[21,66]
Sulfur nanoparticles + silicon dioxide nanoparticles + synthetic fertilizer	Cereals	3.4–45	[65]

Mineral nutrients in NP can be subsidized to plant nourishment in two mechanisms. The initial mechanism is nano structured elements combined in a transporter matrix it may or may not be nano material. For instance, NF of important minerals combined in a matrix such as chitosan, polyacrylic acid, clay or zeolite. In other cases, a nano-structured element in the form of suspension or encapsulated form can directly apply to the plant [7].

The above mentioned two types of nanofertilizers have several advantages over conventional fertilizers such as better solubility and fast absorption or lower leaching. However, the first type of NF is better, as it offers superior control over the speed and timing required for the distribution of the minerals to the plants [32]. There are different classes of NP, which can be transported through endocytosis, can be absorbed by the root, moving through the apoplast and symplast pathways to the xylem, by crossing the endodermis and moving to the rest of the plant through the vascular bundles [32]. Through catering assessed nutrition, the NF can assist the plant production to fight several types of stress (biotic and abiotic). The NF supports improving nutrient efficiency without any side effects [8].

Nutritional quality and tolerance of plants to specific stress are directly influenced by the availability of the required mineral elements. Stress tolerant plants synthesis specific bioactive compounds linked with the specific reaction for antioxidants or osmotic tolerance to different environmental conditions [67].

The properties of NF at minimum intensities are usually optimistic, that increased plant tolerance for abiotic stresses, various pathogens or pests, the metabolic reactions rate, the quantity of antioxidants, and the quality and/or quantity of the harvests. NP and NM at certain quantities bring oxidative tension mediated by the reaction between the NP and NM with various macromolecules like proteins, membranes, nucleic acids due to the presence of lone electrons on the surface of the NP and NM [68].

The NP prepared by the metals is reported to show the oxidative stress in bacteria, fungi, algae, and aquatic and terrestrial plants based on the applied dose. However, this oxidative stress in the plants do not exceed the toxic threshold and not lead to cell death. At the same time the defense mechanism was also activated. In addition to decreasing the price of transportation and field application, NF in small quantity has its own advantage to minimize the cumulation of salt in soil and optimize moisture content enable the plants to fight many biotic and a biotic stresses [68].

## 6 Nanofertilizers vs conventional bulk fertilizers

Both conventional and NF are playing pivotal roles for enhancing the crop yield and nutritional quality by increasing soil fertility [69]. However, nano-structured materials have higher N use efficiency as plants cell walls contain very minute pore sizes (5–20 nm), this leads to the grater nutrient endorsement and improve plants growth (Table 7). This plant developed disease resistance by developing the anti-bending and deep rooting of plants [3].

**Table 7:** Comparisons of nanofertilizers vs conventional fertilizers.

Properties	Nano fertilizer	Conventional fertilizer	References
Solubility	Higher	Lower	[70,71]
Dispersion of mineral nutrients	Advanced spreading of insoluble nutrients	Decrease solubility due to large particle size	
Soil absorption and fixation	Diminished	Elevated	
Bioavailability	High-level	Low-slung	
Effectiveness of Nutrients Uptake	Increased Uptake ratio: saves fertilizer resources. Protecting environmental pollution	Not available to roots and the nutrients uptake efficiency is low loss of materials	
Controlled release	Release rate and pattern precisely controlled	Excess release and leading to toxicity and soil imbalance	
Effective duration of release	Extended effective duration	Used by the plant at the site by the time of application: the rest is converted to insoluble form	
Loss rate	Reduced lose fertilizer nutrients	High lose rate due to leaching, drifting, run off	
Productivity	17–54 % more productive	Comparatively Less	

**Fig. 4:**
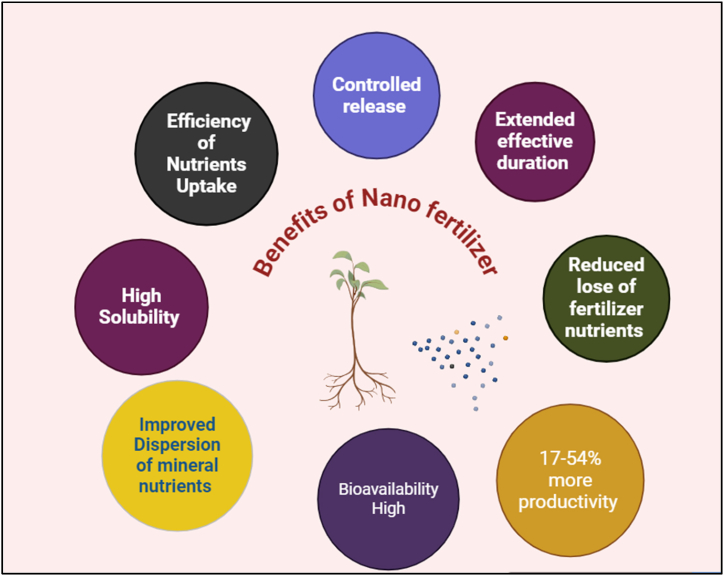
Diagram illustrates the benefits of Nano Fertilizer.

NF has greater benefits over conservative fertilizers (Fig. 4). They contribute to the rise of soil fertility; quality attributes of the products and plants get the tolerance to both biotic and abiotic stress. The researchers established NF positive attributes like nontoxic to nature, environment and humans. In addition, NF reduces the fertilizer input cost and maximizes the profit by higher yields (Table 7) [3].

The other important benefit of the NF is minimizing the accumulation of salt in soil. When compared to conventional pesticides, NF is necessary in little quantities and able to decrease the cost of transportation and field application in addition to the labor force. Therefore, NF is three-times nutrients efficient, around 80–100 times less utilized in compared to chemical fertilizers. The crops are 10 times higher stress tolerant, environmentally friendly, 30 % more nutrient mobilized by the plants, and 17–54 % enhancement in the crop harvest and the superiority of the yields. For example, optimum use of iron NP reported the greater protein content in wheat [[69], [70], [72]]

## 7 Current applications of nanofertilizers

Technological progress and modernizations have been achieved in contemporary times in the agriculture sector and particle engineering. The advances explain distinctive targeted features with raised strength, particularly, NT has possible to offer the actual results to the numerous agriculture-related problems [73]. Extensive use of agrochemicals such as herbicides, fungicides, insecticides, and chemical fertilizers have reported hostile environmental and community influences, as well as trigger conflict in target species [74].

NF are one of the recent potential outputs, in a significant break for agriculture which plays the main part in the physiological and biochemical cycle of crops through the availability of nutrients with substantial surface area. The lower size of NM allows expanded relations and efficient nutrient understanding for crop fertilization [73].

NF manufactured (Table 8) with the precise intension to regulate the release of nutrients. This control liberation enhances the yield and the nutrient use efficiently and minimizes the hammering of nutrients. These attributes assistance to conquer the chronic difficulty of eutrophication, which is an exceptionally unique feature of NP such as eminent surface area or capacity size proportion and better the physicochemical attributes, associated to their majority complements. Hence, NF is now appearing as a favorable approach to further plant growth and yields [75].

**Table 8:** Some commercial Nano fertilizers manufacturing agents.

Commercial Nano fertilizers	Major Constitutes	Manufacturer
Nano Ultra-Fertilizer 500g	Organic matters (5.5 %), Nitrogen (10 %), P_2_O_5_ (9 %), K_2_O (14 %), P_2_O_5_ (8 %), MgO (3 %)	SMTET Eco-Technologies Co, Ltd., Taiwan
Nano calcium (Magic green) 1 kg	CaCO_3_ (77.9 %), MgCO_3_ (7.4 %), SiO_2_ (7.47 %), K (0.2 %), Na (0.03 %), P (0.02 %), Fe(7.4 ppm), Al_2_O_3_ (6.3 ppm), Sr (804 ppm), Sulphate (278 ppm), Ba (174 ppm), Mn (172 ppm), Zn (10 ppm).	AC International Network Co., Ltd., Germany
Nano capsule	N (0.5 %), P_2_O_5_ (0.7 %), K_2_O (3.9 %), Ca (2.0 %), Mg (0.2 %), S (0.8 %), Fe (2.0 %), Mn (0.004 %), Cu (0.007 %), Zn (0.004 %).	The Best International Network Co., Ltd., Thailand
Nano micronutrient (Eco star) 500g	Zn (6 %), B (2 %), Cu (1 %), Fe (6 %), EDTA, Mo (0.05 %), Mn (5 %), AMINOS (5 %)	Shan maw Myae Trading Co., Ltd., India
PPC Nano (120) mL	Mprotein (19.6 %); Na_2_O (0.3 %); K_2_O (2.1 %), (NH_4_)_2_SO_4_ (1.7 %), Diluent (76) %	WAI International Development Co., Ltd., Malaysia
Nano max NPK fertilizer	Multiple organic acids chelated with major nutrients, amino acids, organic carbon, organic micronutrients/trace elements, vitamins, and probiotic	JU Agri Sciences Pvt. Ltd., Janakpuri, New Delhi, India
TAGNANO (NPK, Pho, S, Zinc, Cal, etc.) fertilizers	Proteino-Lacto-Gluconate Chelated with Micronutrients, Vitamins, probiotic, seaweeds, extracts and humic acid	Tropical Agro system India(P) Ltd., India
Nano Green	Extracts of corn, grain, soybean, potatoes, coconut and palm	Nano green Sciences, Inc., India
Biozar Nano fertilizer	Combination of organic Materials, micronutrients	Fanavar Nano-Pazhoohesh Markazi campani, Iran

Nano technology is powerful technology to transform the agricultural and food industry by formulating nanofertilizers with new tools for improving the capability of plants to engross nutrients, smart devices and smart distribution systems that help agricultural commerce to fight against viruses and other crop pathogens [71,75]. NF for efficient nutrient management fashioned with the help of NT can be subjugated the supply chain of whole agriculture system; fertilizer plays an essential role in cumulating the agricultural; but extreme practices of fertilizers permanently modify the chemical ecology of soil and further plummeting the accessible area for crop production. Formulation of NF is the greatest significant field of agriculture which has been fascinating consideration of the soil scientists as well as the ecologists because of its ability to upsurge harvest, advance soil fertility, reduce pollution, and make a promising environmental microflora (Henry Sukirtha and Saranya, 2020).

## 8 Limitation and prospects of nanofertilizers

NF are designed and formulated as prime agricultural contributions currently applied in many agriculture sectors to improve agricultural food productivity. However, questions are raising completed potential human and environmental impacts. Even though experts’ opinion indicates that food products comprising NP obtainable in the market are possibly safe to eat, the thoughtful introducing of nano-sized ingredients in agricultural activities is very importent (35; 75).

The usage of NP as fertilizers is thoroughly being-explored, before applied in agriculture or farming practice. NP with high concentration may be exposed through nano food and believed to be of prospective risk for human. Especially, widespread release of nanomaterials into the environment and the food chain may pose a risk to human health because all NM may not be equally safe for all applications. Therefore, due to the great possibility to enter human circulation and react with the vital organs, depth scientific toxicological studies in both *in vivo* and *in vitro* should be conducted to secure safety to the environmental and human health.

The other limitation of using nano material may require highly qualified professionals, and it is too costly to establish production set ups. However, agriculture is continuously most potential and steady segment that should be supported though use of NT with materials science and biomass alteration methods which functional in agriculture as the base of delivering food, to attain the challenges of food security and environmental protection [76].

## 9 Conclusions

NF are applied in many agricultural systems with organic provisions to reduce environmental pollution by staggered and gradual released of fertilizers. Coating or encapsulation of NP with organic or inorganic resources in the limited rate, and extent of plant nutrient. The outlook of NF for sustainable crop invention and universal adaptation is based on numerous factors such as proper regulations and synthesis of novel NF based on the requirements. However, application of NP should be prudently intended and inclusive toxicity test concerning both *in vivo* and *in vitro* been supplied out to assure protection to the environmental and human well-being because of all NM may not be similarly safe for all applications. Deliberately introducing nano-sized materials within high concentration may expose nano-food and potential risk to human health particularly, wide proclamation of nontoxic materials into the environment and the food chain may result in unintended health outcomes. The limitation of using nano material also may require highly qualified professionals and higher cost in establishment of production setups.

## CRediT authorship contribution statement

**Birara Melku Ayenew:** Writing – review & editing, Writing – original draft, Resources, Methodology, Investigation, Formal analysis, Data curation. **Neela Satheesh:** Writing – review & editing, Writing – original draft, Methodology, Conceptualization. **Zemenu Birhan Zegeye:** Supervision, Resources, Formal analysis, Data curation. **Desalegn Adisu Kassie:** Writing – review & editing, Writing – original draft, Resources, Methodology, Data curation.

## Disclosure statement

None of the authors are not reported any conflict of interest.

## Data and Code availability statement

No data was used for the research described in the article.

## Ethical approvals

Not applicable.

## Funding

No funding was grant for this work.

## Declaration of competing interest

The authors declare that they have no known competing financial interests or personal relationships that could have appeared to influence the work reported in this paper.

## References

[1] Bakker T., Brauers E., van der Elst G., Wangari E. (2023). Nanotechnology in agricultural production nanotechnology in agricultural production. A policy brief was commissioned by the UN policy analysis branch. Division for Sustainable Development.

[2] El-Henawy A., El-Sheikh I., Hassan A., Madein A., El-Sheikh A., El-Yamany A., Radwan A., Mohamed F., Khamees M., Ramadan M., Abdelhamid M., Khaled H., El-Faramawy H., Ayoub Y., Youssef S., Faizy S.E.-D. (2018). Response of cultivated broccoli and red cabbage crops to mineral, organic and nano-fertilizers. Environment. Biodiversity and Soil Security.

[3] El-Ramady H., El-Ghamry A., Mosa A., Alshaal T. (2018). Nanofertilizers vs. Biofertilizers: new insights. Environment. Biodiversity and Soil Security.

[4] Smith L.E., Siciliano G. (2015). A comprehensive review of constraints to improved management of fertilizers in China and mitigation of diffuse water pollution from agriculture. Agric. Ecosyst. Environ..

[5] Kumar Y. (2020). Nanofertilizers for enhancing nutrient use efficiency, crop productivity and economic returns in winter season crops of Rajasthan. Annals of Plant and Soil Research.

[6] Li Wei-Qi, Qing Ting, Li Cheng-Cheng, Li Feng, Ge Fei, Fei Jun-Jie, Willie J., Peijnenburg G.M. (2020). Integration of subcellular partitioning and chemical forms to understand silver nanoparticles toxicity to lettuce (*Lactuca sativa* L.) under different exposure pathways. Chemosphere.

[7] Dimkpa C.O., Bindraban P.S. (2017). Nanofertilizers: new products for the industry?. Journal of Agriculture and Food Chemistry.

[8] Dwivedi S., Saquib Q., Al-Khedhairy A.A., Musarrat J., Dwivedi (2016). Microbial Inoculants in Sustainable Agricultural Productivity.

[9] Claudia P., Mauro V., Emilio R.C. (2014). https://ideas.repec.org/p/ipt/iptwpa/jrc89736.html.

[10] Kumar S., Jasmin L., Saravaiya S. (2018).

[11] Siddiqui M.H., Al-Whaibi M.H., Mohammad F. (2015). Nanotechnology and Plant Sciences: Nanoparticles and Their Impact on Plants.

[12] Kookana R.S., Boxall A.B.A., Reeves P.T., Ashauer R., Beulke S., Chaudhry Q., Cornelis G., Fernandes T.F., Gan J., Kah M., Lynch I., Ranville J., Sinclair C., Spurgeon D., Tiede K., Van Den Brink P.J. (2014). Nanopesticides: guiding principles for regulatory evaluation of environmental risks. J. Agric. Food Chem..

[13] Kalra T., Tomar P.C., Arora K. (2020). Micronutrient encapsulation using nanotechnology : nanofertilizers. Plant Archives.

[14] Savci S. (2012). Investigation of effect of chemical fertilizers on environment. Apcbee Procedia.

[15] Kim M.J., McNally B., Murata K., Meller A. (2007). Characteristics of solid-state nanometre pores fabricated using a transmission electron microscope. Nanotechnology.

[16] Hossain A., Kerry R.G., Farooq M., Abdullah N., Tofazzal Islam M., Devarajan (2020). Nanotechnology for Food, Agriculture, and Environment.

[17] Saraiva R., Ferreira Q., Rodrigues G.C., Oliveira M. (2022). Phosphorous nanofertilizers for precise application in rice cultivation as an adaptation to climate change. Climate.

[18] Muhammad M., Khalid M.K., Marcela C.C., Lucas B.C., Leonardo F.F., Rania E.M., Maher Z.E., Murat K., Jalel L., Hidayat U., Depeng W. (2020). Chitosan-based delivery systems for plants: a brief overview of recent advances and future directions. Internationa Journal of Biolological Macromolecules.

[19] Monreal C.M., Derosa M., Mallubhotla S.C., Bindraban P.S., Dimkpa C. (2016). Nanotechnologies for increasing the crop use efficiency of fertilizer-micronutrients. Biol. Fertil. Soils.

[20] Rech I., Polidoro J.C., Pavinato P.S. (2017). Additives incorporated into urea to reduce nitrogen losses after application to the soil. Pesqui. Agropecuária Bras..

[21] Yang M., Fang Y., Sun D., Shi Y. (2020). Efficiency of two nitrification inhibitors (dicyandiamide and 3, 4-dimethypyrazole phosphate) on soil nitrogen transformations and plant productivity: a meta-analysis. Sci. Rep..

[22] Diatta J., Borowiak K., Szczepaniak W. (2018). Evaluation of fertilizers solubility and phosphate release in slightly acidic arable soil. Arch. Agron Soil Sci..

[23] Morales-Díaz A.B., Ortega-Ortíz H., Juárez-Maldonado A., Cadenas-Pliego G., González-Morales S., Benavides-Mendoza A. (2017). Application of nanoelements in plant nutrition and its impact in ecosystems. Adv. Nat. Sci. Nanosci. Nanotechnol..

[24] Suppan S. (October 2017).

[25] Eroglu N., Emekci M., Athanassiou C.G. (2017). Applications of natural zeolites on agriculture and food production. J. Sci. Food Agric..

[26] Polat E., Karaca M., Demir H., Onus N. (2004).

[27] Selva P., Balakrishnan N. (2017). A review of nano fertilizers and their use and functions in soil. International Journal of Current Microbiology and Applied Sciences.

[28] Monostori T. (2014).

[29] Duan Q., Jiang S., Chen F., Li Z., Ma L., Song Y., Xuejun Y., Yongxin C., Hongsheng L., Yu L. (2023). Fabrication, evaluation methodologies and models of slow-release fertilizers: a review. Ind. Crop. Prod..

[30] Pickering H.W., Menzies N.W., Hunter M.N. (2002). Zeolite/rock phosphate—a novel slow-release phosphorus fertiliser for potted plant production. Sci. Hortic..

[31] Vázquez-Núñez E., López-Moreno M.L., de la Rosa Álvarez G., Fernández-Luqueño F. (2018). Agricultural Nanobiotechnology: Modern Agriculture for a Sustainable Future.

[32] Ijaz I., Gilani E., Nazir A., Bukhari A. (2020). Detail review on chemical, physical and green synthesis, classification, characterizations and applications of nanoparticles. Green Chem. Lett. Rev..

[33] HobAllah E., Saber M., Zaghloul A. (2019). Nanotechnology applications in agriculture. Int. J. of Environmental Pollution & Environmental Modelling.

[34] Jahangirian H., Rafiee-Moghaddam R., Jahangirian N., Nikpey B., Jahangirian S., Bassous N., Saleh B., Kalantari K., Webster T.J. (2020). Green synthesis of zeolite/Fe2O3 nanocomposites: toxicity & cell proliferation assays and application as a smart iron nanofertilizer. Int. J. Nanomed..

[35] Hussein H.S., Shaarawy H.H., Hussien N.H., Hawash S.I. (2019). Preparation of nano-fertilizer blend from banana peels. Bull. Natl. Res. Cent..

[36] Subbaiya R. (2012). Formulation of green nano-fertilizer to enhance the plant growth through slow and sustained release of nitrogen. J. Pharm. Res..

[37] Li J., Wu Q., Wu J., Mahmood A. (2015). Handbook of Nanoparticles.

[38] Sivasankaran S., Sankaranarayanan S., Ramakrishnan S. (2013). Materials Science Forum.

[39] Ziylan Y.A., Mizukoshi Y., Maeda Y., Ince N.H. (2015). Supporting pristine TiO_2_ with noble metals to enhance the oxidation and mineralization of paracetamol by sonolysis and sonophotolysis. Appl. Catal. B Environ..

[40] Perez, T. E, Pinilla, M. G., Mejia, R.S, Ortiz, M.U, Torres, A., Jose, Y.M. (20008). Highly size-controlled synthesis of Au/Pd nanoparticles by inert-gas condensation. Faraday Discuss, 138, 353-362. 10.1039/B705913M. 18447025

[41] Banerjee A.N., Krishna R., Das B. (2008). Size controlled deposition of Cu and Si nano-clusters by an ultra-high vacuum sputtering gas aggregation technique. Appl Physics A.

[42] Konrad A., Herr U., Tidecks R., Kummer F., Samwer K. (2001). Luminescence of bulk and nanocrystalline cubic yttria. J. Appl. Phys..

[43] Andrievskii R.A. (1994). The synthesis and properties of nanocrystalline refractory compounds. Russian Chemical Reviews.

[44] Shanmu D., Xiao C., Lin G., Lixue Z., Xinhong Z., Zhihong L., Pengxian H., Hongxia X., Jian Y., Xiaoying Z., Lanfeng L., Chaoqun S., Guanglei C. (2011). A biocompatible titanium nitride nanorods derived nanostructured electrode for biosensing and bio-electrochemical energy conversion, 26 (10). Biosens. Bioelectron..

[45] Boddolla S., Thodeti S. (2018). A review on characterization techniques of nanomaterials. International Journal of Engineering, Science and Mathematics.

[46] Rastogi A. (2017). A mini-review practice of formulations of nanoparticles. International Journal of Chemical Synthesis and Chemical Reactions.

[47] Dikusar A.I., Globa P.G., Belevskii S.S., Sidel’nikova S.P. (2009). On limiting rate of dimensional electrodeposition at meso-and nanomaterial manufacturing by template synthesis. Surf. Eng. Appl. Electrochem..

[48] Ponon N.K., Appleby D.J., Arac E., King P.J., Ganti S., Kwa K.S., Anthony O.N. (2015). Effect of deposition conditions and post-deposition anneal on reactively sputtered titanium nitride thin films. Thin Solid Films.

[49] Guler U., Shalaev V.M., Boltasseva A. (2015). Nanoparticle plasmonics: going practical with transition metal nitrides. Mater. Today.

[50] Iravani S. (2011). Green synthesis of metal nanoparticles using plants. Green Chem..

[51] Pandey S., Oza G., Mewada A., Sharon M. (2012). Green synthesis of highly stable gold nanoparticles using momordicacharantia as nano fabricator. Arch. Appl. Sci. Res..

[52] Manjula P., Boppella R., Manorama S.V. (2012). A facile and green approach for the controlled synthesis of porous SnO2 nanospheres: application as an efficient photocatalyst and an excellent gas sensing material. ACS Appl Materials Interfaces.

[53] Vidya C., Hiremath S., Chandraprabha M.N., Antonyraj M.L., Gopal I.V., Jain A., Kokil B. (2013). Green synthesis of ZnO nanoparticles by *Calotropis gigantea*. International Journal of Current Engineering and Technology.

[54] Aboul-Enein A.M., Salama Z.A., Gaafar A.A., Aly H.F., Bou-Elella F.A., Ahmed H.A. (2016). Identification of phenolic compounds from banana peel (*Musa paradaisica* L.) as antioxidant and antimicrobial agents. J. Chem. Pharmaceut. Res..

[55] Yaseen R., I. S. Ahmed A., Mohamed A.K.M., Agha M., Emam T. (2020). Nano-fertilizers: bio-fabrication, application and biosafety. Novel Research in Microbiology Journal.

[56] Jakkula V.S., Wani S.P. (2018). Zeolites: potential soil amendments for improving nutrient and water use efficiency and agriculture productivity. Scientific Reviews & Chemical Communications.

[57] Sharma V., Javed B., Byrne H., Curtin J., Tian F. (2022). Zeolites as carriers of nano-fertilizers: from structures and principles to prospects and challenges. Applied Nano.

[58] Manikandan A., Subramanian K. (2016). Evaluation of zeolite-based nitrogen nano-fertilizers on maize growth, yield and quality on inceptisols and alfisols. International Journal of Plant & Soil Science.

[59] Kaushik G., Vishnu J., Arslan R. (2014). Manufacture and categorization of nanoporous zeolite based N fertilizer. African Journal of Agronomy.

[60] Petosa A.R., Rajput F., Selvam O., Ohl C., Tufenkji N. (2017). Assessing the transport potential of polymeric nanocapsules developed for crop protection. Water Res..

[61] Kottegoda N., Munaweera I., Madusanka N., Karunaratne V. (2011). A green slow-release fertilizer composition based on urea-modified hydroxyapatite nanoparticles encapsulated wood. Current science.

[62] Salam A., Khan A.R., Liu L., Yang S., Azhar W., Ulhassan Z., Zeeshan M., Wu J., Fan X., Gan Y. (2022). Seed priming with zinc oxide nanoparticles downplayed ultrastructural damage and improved photosynthetic apparatus in maize under cobalt stress. J. Hazard Mater. Part A.

[63] Balouch A., Agheem M.H., Ahmed S., Baloch A.R., Tunio A., Hameed A. (2020). Efficient mitigation of cadmium and lead toxicity in coriander plant utilizing magnetite (Fe_3_O_4_) nanofertilizer as growth regulator and antimicrobial International Journal of Environmental Analytical Efficient mitigation of cadmium and lead toxicity. Int. J. Environ. Anal. Chem..

[64] Tarafder C., Daizy M., Alam M., Ali R., Islam J., Islam R., Ahommed S., Aly M., Aly S., Khan Z.H. (2020). Formulation of a hybrid nanofertilizer for slow and sustainable release of micronutrients. ACS Omega.

[65] Zhang D., Ma X.L., Gu Y., Huang H., Zhang G.W. (2020). Green synthesis of metallic nanoparticles and their potential applications to treat cancer. Front. Chem..

[66] Elshayb O.M., Nada A.M., Farroh K.Y., Al-Huqail A.A., Aljabri M., Binothman N., Seleiman M.F. (2022). Utilizing urea–chitosan nanohybrid for minimizing synthetic urea application and maximizing Oryza sativa l. productivity and n uptake. Agriculture.

[67] Sharifi M., Faryabi K., Talaei A.J., Shekha M.S., Ale-Ebrahim M., Salihi A. (2020). Antioxidant properties of gold nanozyme: a review. J. Mol. Liq..

[68] Abdalla N., Ragab M.I., Fári M., El-Ramady H., Alshaal T., Elhawat N., Elmahrouk M., Elzaawely A., Elsakhawy T., Omara A.E.-D., Taha H. (2019). Nanobiotechnology for plants. Environment, Biodiversity and Soil Security.

[69] Banotra M., Kumar A., Sharma B.C., Nandan B., Verma A., Kumar R., Gupta V., Bhagat S. (2017). Prospectus of use of nanotechnology in agriculture–A review. International Journal of Current Microbiology and Applied Sciences.

[70] Kandil E.E., Marie E.A.O. (2017). Response of some wheat cultivars to nano- , mineral fertilizers and amino acids foliar application. Alexandria Science Exchange Journal: An International Quarterly Journal of Science Agricultural Environments.

[71] Cui H.X., Sun C.J., Liu Q., Jiang J., Gu W. (2010). *Proceedings of the International Conference on Nanoagr*i, Sao Pedro, Brazil, 20–25 June 2010.

[72] Raliya R., Saharan V., Dimkpa C., Biswas P. (2018). Nanofertilizer for precision and sustainable agriculture: current state and future perspectives. J. Agric. Food Chem..

[73] DeRosa M.C., Monreal C., Schnitzer M., Walsh R., Sultan Y. (2010). Nanotechnology in fertilizers. Nat. Nanotechnol..

[74] SánchezB. F., Sánchez B. F, van den B. P.J., Mann R.M. (2011). Ecological Impacts of Toxic Chemicals.

[75] Henry Sukirtha T., Saranya N. (2020). Applications of nanotechnology in agricultural waste–review paper. Scholars Acad. J. Biosci..

[76] Camara M.C., Campos E.V.R., Monteiro R.A., Do Espirito Santo Pereira A., De Freitas Proença P.L., Fraceto L.F. (2019). Development of stimuli-responsive nano-based pesticides: Emerging opportunities for agriculture. J. Nanobiotechnol..

